# Design and Implementation of IoT Data-Driven Intelligent Law Classroom Teaching System

**DOI:** 10.1155/2022/8003909

**Published:** 2022-03-25

**Authors:** Gaoyan Shi

**Affiliations:** Shijiazhuang Vocational and Technical College, Shijiazhuang 050081, China

## Abstract

In this paper, we conduct in-depth research and analysis by building an IoT data-driven intelligent law classroom teaching system and implementing it in the actual teaching process. Firstly, the application requirements and main functions of the classroom interactive system are analysed and studied in depth; the overall design of the classroom interactive system based on wireless communication is carried out; and the classroom interactive system consisting of teacher's receiver, wireless receiver, student's handheld terminal, teacher's receiver upper computer software, student's handheld terminal upper computer software, and data management website is developed. Platform and communication part: middleware comprises multiple modules such as real-time memory event database, task management system, and event management system. The system uses USB communication, serial time-sharing communication, Zigbee wireless communication, WebSocket, and other technologies to realize data communication between several modules, and some key technologies are studied and improved in detail. Finally, the system is deployed to the actual experimental application scenario, and the software and hardware construction of the IoT innovation lab is completed. The lab equipment is officially put into use, the information platform is also in the trial run stage, and the system is tested in time during the operation stage to provide the basis for the improvement of the system functions. Use data to conduct an in-depth analysis of learning situations and learning process analysis, optimize the reasonable construction of a personalized learning environment, serve personalized learning, and achieve the most optimal learning effect. The secondary development of the Turing robot provides a personalized auxiliary Q&A system for teaching, allowing artificial intelligence-assisted teaching to be integrated into the curriculum. With the help of teaching law courses, the empirical study demonstrates that the personalized learning environment has significant advantages in improving teaching effectiveness.

## 1. Introduction

The rapid development of information technology has accelerated the deep integration of information technology and education teaching, information technology platforms, and artificial intelligence-assisted teaching; other information technology resources have emerged; and a variety of new learning methods have emerged to show that education is making headway towards the information technology process. However, due to the complexity of information tools, teaching reform often tends to go astray, and very often information technology tools become just a landmark application to highlight new learning styles, staying only for information technology competitions, or open or observed classes, and not falling into the classroom reality for students [1]. The main research purpose of this paper is to select, integrate, and develop informatization resources under a data-driven perspective and build a personalized informatization learning environment that meets the school, teaching, and learning conditions. Network nodes are required to have the self-adjustment ability, self-adaptation ability, and robustness and complete tasks such as network initialization and fault self-repair through topology control mechanisms, network protocols, and cooperation to maintain the normal operation of the network. Traditional school education cannot teach each student according to his or her ability, but the information age has brought new education opportunities [2]. With artificial intelligence and big data, the whole process of student learning can be monitored and tracked in real time, and the problems and deficiencies of students can be analysed so that teachers can tailor their learning plans to the specific situation of students. The emphasis on process-oriented data on student learning guides students to adjust their learning styles promptly, making large-scale personalized learning possible. While offline traditional education allows teachers to impart knowledge and solidify the foundation, online education breaks the time and space constraints from which students can access personalized resources and learning styles.

Changing the traditional education model through new educational technology equipment has received increased attention from schools [3]. At present, the information management system for teachers and students has been widely used in colleges and universities, realizing the functions of integrated management of teacher and student information, online course selection, online grade entry and query, and integrated query of school information. In particular, the online grade entry and query function of students is especially widely used among teachers and students. However, the current teaching situation in domestic universities is still the traditional irrigation method interspersed with multimedia teaching methods such as PPT. Teachers still count students' knowledge proficiency through traditional after-class assignments and randomly checking students' answers to questions in class [4]. At the same time, according to the student's learning data feedback, the learning environment resources are adjusted in real time, and finally, a dynamic and personalized learning environment that conforms to the academic conditions is constructed. Once students do not keep up with the teacher's teaching ideas, it is difficult to listen to the later knowledge. As for the student's attendance, teachers usually still confirm it through the roll call.

About the methodological aspects of rule of law education, rule of law education is most often based on problem-based and case-based approaches [5]. The problem-based approach requires students to identify, construct, and analyse problems, and students, therefore, spend more time on learning activities and perform better than those in the case-based approach. Based on the needs of laboratory management and practical training teaching, it is necessary to research and explore laboratory intelligence and informatization for the management of university laboratories. If the requirements are met, open the serial port communication switch for communication, and close the serial port switch after the communication is completed. If it does not meet the requirements, wait for a period, continue to query until there is no other coordinator communication, and then turn on the serial port switch. In this paper, based on the existing perfect facilities, the structure and characteristics of IoT technology in smart home and smart building are applied to the laboratory management system, exploring the model of intelligent laboratory, constructing IoT innovation training room, and developing smart campus class terminal management system, which integrates the use of IoT, mobile Internet, big data, intelligent perception, and other emerging information technologies, on the basis of traditional campus, to provide timely feedback to parents and teachers on students' safe attendance and abnormal attendance; to organically combine all aspects of home-school interaction, campus information, announcement release, class culture, teacher-student interaction, class walking teaching and teaching management; to build an information management platform for campus and class management and student growth; enrich the overall school information technology environment and information dissemination channels; efficiently and conveniently deliver various knowledge information and notice information; provide timely feedback on classroom information; realize transparent management of classes; and provide strong technical support for the digitization and information of education and teaching in our school.

## 2. Status of Research

The spreading factor and the communication rate are found to have a significant impact on the coverage capability of the LoRa network through field tests. The limitations of the Lora WAN protocol are proposed through the analysis of Lora WAN protocol, the size of the Lora WAN network is much smaller than the theoretical value under the limitation of duty cycle transmission, and the adoption of transmission acknowledgment answering mechanism for reliable transmission also makes the network capacity smaller in the same way [6]. Through different IoT application cases, the advantages and disadvantages of Lora WAN in different scenarios are analysed and its applicability scope is illustrated. The authors argue that the random-access method of Aloha for nodes in Lora WAN networks is not the optimal solution and a more complex TDMA access method can be considered. By modelling and simulating a large Lora WAN network using the NS-3 network simulator, the simulation results show that the limited downlink communication in the Lora WAN network reduces the packet delivery rate of the uplink communication that needs to be acknowledged and adding gateways can improve but not eliminate this effect [7]. A comprehensive analysis of the features and limitations of Lora WAN gives the current problems of the Lora WAN protocol, that is, high data loss rate at high data concurrency and long retransmission time for the timely arrival of data [8]. The physical layer transmission characteristics of the Lora WAN network are analysed, the packet loss rate and conflict probability of different data frames at the gateway are derived by building a signal reception model, and the variation of packet loss rate and conflict probability with different spreading factors and sending cycles are illustrated by simulation results [9].

With the development of communication technology, classroom interactive systems have gradually entered university campuses and are playing an increasingly important role in student learning. Multimedia teaching has also become a standard part of daily university teaching activities [10]. Many university research project groups and some social research institutions at home and abroad have also conducted a series of research on education informatization [11]. There are currently two ideas for developing classroom interactive systems. One idea is to upgrade the hardware facilities completely and transform them into intelligent classrooms [12]. However, these devices require a large-scale and comprehensive upgrade of the classroom hardware; the amount of engineering, time costs, and transformation costs are relatively large. Moreover, if a certain aspect needs to be upgraded at a later stage, it will be more difficult [13]. The data management web terminal is mainly used to bind the user's school number and the MAC address of the student's handheld terminal. The teacher downloads the student list and the corresponding MAC address list of the class to be taught, stores and analyses the students' classroom answer and question data collected by the teacher, and visualizes these data. For the student user, it can display the students' usual classroom performance in real time and show it in a chart; for the teacher user, it can display his classroom performance in real time [14]. For teachers, they can display the class performance of students in their classes in real time and display it in charts [15].

With the continuous development and updating of computer technology, big data, cloud computing, and artificial intelligence will be widely used in all areas of social life. For example, big data can be used to predict the occurrence of crimes; companies can analyse the sales of products based on customer reviews on shopping websites; the National Centre for Disease Control and Prevention can analyse the spread of diseases based on Internet users' search records; and so on [16]. For the large amount of data generated, the integration and analysis of the data allow people to discover new knowledge and create new value. As the volume of IoT business increases, the amount of data storage and computation will bring the need for “computing” power to the cloud. Cloud computing will provide the appropriate services in infrastructure, platforms, and software. Artificial intelligence is a branch of computer science and new technical science that combines the application of computers and other disciplines, which focuses on the study of theories, methods, and technologies to simulate, extend, and expand human intelligence [9].

## 3. IoT Data-Driven Intelligent Law Classroom Teaching System Design

### 3.1. IoT Smart Classroom Teaching System Design

There is no doubt that the communication among many nodes in IoT involves addressing the problem, and IPv4 addresses are getting scarce. Its address space can hardly meet the demand for address space in today's IoT. Moreover, IPv4 has a shortcoming for the mobility of the Internet, and the MIPv4 mechanism proposed in the IETF to support the mobility of nodes will lead to rapid consumption of network resources for many nodes, resulting in network paralysis [17]. IPv6 technology not only has a huge address space but also adopts a stateless address allocation scheme to solve the problem of allocation efficiency of massive addresses. For node mobility, IPv6 proposes the concept of IP address binding buffer and introduces a method to detect node movement. At the same time, IPv6 design considers the quality of service, such as defining the traffic class field and flow label field in the packet structure and using the flow label carried by the packet to do a detailed division of services. It is a protocol designed for communication between remote sensors and control equipment that have limited computing and storage resources and work on low-bandwidth, unreliable networks. It has the characteristics of simplicity, strong scalability, and low traffic.

**Figure 1:**
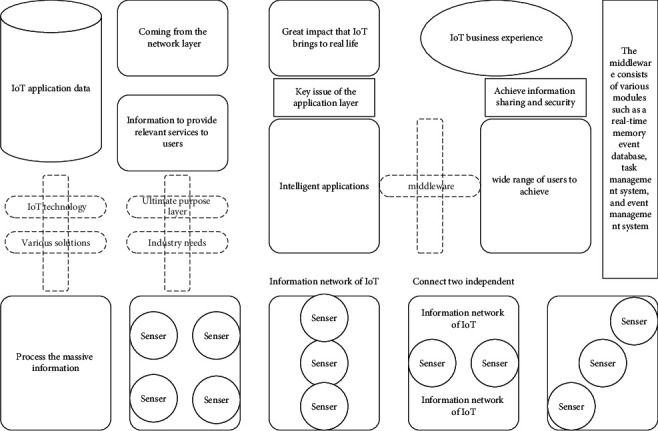
IoT framework design.

The IoT application layer is the ultimate purpose layer, which is the combination of IoT and industry needs, by docking IoT technology with industry technology and using various solutions to provide a good IoT business experience for a wide range of users to achieve a wide range of intelligent applications, so that people can feel the great impact that IoT brings to real life. The main function of the IoT application layer is to process the massive information coming from the network layer and use this information to provide relevant services to users. The key issue of the application layer is how to achieve information sharing and security. Among them, the rational use, as well as efficient processing of relevant information, is an urgent IoT problem, and to solve this technical challenge, the IoT application layer needs to utilize technologies such as middleware, Machine to Machine (M2M), and edge cloud computing. In building the information network of IoT, middleware is mainly applied to distributed systems to connect various technologies and share resources. Middleware can be divided into two parts: the platform and the communication part. The middleware consists of various modules such as a real-time memory event database, task management system, and event management system, which can be implemented to connect two independent applications, as shown in Figure 1.

It is a protocol designed for communication between remote sensors and control equipment that have limited computing and storage resources and work on low-bandwidth, unreliable networks. It has the characteristics of simplicity, strong scalability, and low traffic. A wireless sensor network consists of tiny nodes deployed in the monitoring area with data processing units, miniature sensors, and communication modules through a self-organizing wireless self-organizing network system. With the help of the nodes' built-in sensors measuring the signals of the objects around their location, the observer can obtain information about the sensed objects in the network coverage area. The three elements of wireless sensor networks are sensor, sensing object, and observer [18]. The characteristics of wireless sensor networks are mainly reflected in the size of scale and the purpose of application. In the application of a wireless sensor network, the location of sensor nodes cannot be set precisely in advance. Therefore, the network nodes are required to have self-regulation ability, self-adaptive ability, and robustness to maintain the normal operation of the network through topology control mechanism and network protocols and mutual collaboration to complete such work as network initialization and fault self-healing. The basic structure of wireless sensor networks can be divided into node types, node structures, and function-based models of wireless sensor network structures. Its technical research is to be mainly focused on medium access control protocols, network topology control, routing protocols, node positioning, clock synchronization, data management, data fusion, embedded operating systems, and network security.

Based on the in-depth analysis of constructivist theory, we propose the theory of realm pulse learning and build a personalized learning environment with an information platform and AI teaching assistance. It is a new paradigm to guide the transformation of learning, which allows the interaction between the many learning elements of the learner and the external elements of the teacher's guidance to form a self-renewal force. In many cases, informatization methods have only become an iconic application for demonstrating new ways of learning. They only serve for informatization competitions or open classes and observation classes and have not been implemented in the classroom to serve students. The AI-assisted teaching of the information platform can undoubtedly provide a better learning environment for the student-cantered teaching model while adjusting the learning environment resources in real time based on the feedback of students' learning data, finally building a dynamic and personalized learning environment in line with the learning situation.

**Figure 2:**
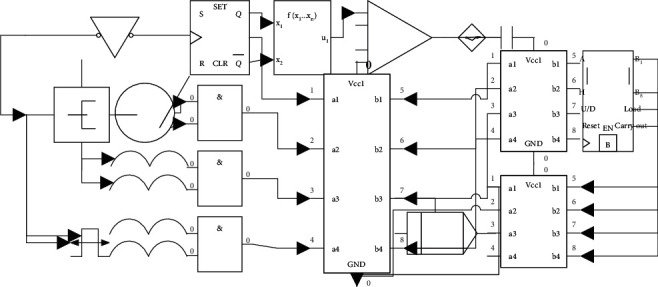
Time-sharing communication serial port principle.

Then, to make data-driven decision-making applied to teaching in addition to the basic elements, some conditions are needed. One is based on the concept of big data teaching, education data as a practical tool, and means to promote the improvement and perfection of teaching continuously. Second, for the correct understanding of the application of data in teaching, the big data should have a cautious attitude; there may be some personal privacy, data security, and other issues; and we should adhere to the principle of science and objectivity, combined with the actual situation and flexible choice. The third is the construction of an effective platform for data collection and application, to be able to use the platform to carry out teaching activities inside and outside the classroom, collect learning data, carry out learning tracking, and make data decisions truly applied to teaching, as shown in Figure 2.

To ensure that more student handhelds can be connected in a wireless-based classroom interactive system, we need a teacher receiver and 4 Zigbee coordinators to transmit information to each other. Reasonably use educational data to customize a personalized learning environment to meet the learning needs of students at different levels so that artificial intelligence and big data can truly promote more personalization, thereby increasing the attractiveness of the classroom. The teacher receiver uses one serial port to connect to 4 wireless receivers, respectively. At the same time, there are 4 LCD lights connected to each hardware channel to show which coordinator of the wireless receiver the teacher receiver is communicating with. There is also a serial communication switch connected to each serial path, which can only be communicated when the switch is closed. When the teacher needs to send a message to the 4 serial ports with the receiver, the 4 serial communication switches can be turned on and off in sequence to ensure that only one serial communication switch is on for each communication. When a coordinator needs to send a message to the teacher receiver, it also needs to make sure no other coordinator is communicating first. If the requirements are met, then turn on the serial communication switch to communicate and turn off that serial switch when the communication is complete. If the requirement is not met, then wait for some time and continue to query until no other coordinator is communicating before turning on the serial switch.

Reasonably use educational data to customize a personalized learning environment to meet the learning needs of students at different levels so that artificial intelligence and big data can truly promote more personalization, thereby increasing the attractiveness of the classroom. A typical Lora WAN network architecture consists of four parts: nodes, gateways, web servers, and application servers. The nodes are mainly responsible for the collection and reporting of sensing data and the reception and execution of command data on the server side. The nodes adopt the ALOHA access method to randomly access the network and send data on demand. The data reported by a node can be received by multiple gateways, thus forming a star network topology with the gateways, which can ensure the reliability of the connection and realize the access of a larger number of devices in the network. The gateway is the relay device between the node and the network server in the Lora WAN network as the central node of the star network, forwarding the LoRa RF data from/to the node and TCP/P network data for processing. The network server is an important part of the Lora WAN network for data processing and device access, responsible for LoRa node data parsing and encapsulation, encryption, decryption, LoRa node entry authentication, Adaptive Date Rate (ADR) regulation, and other functions. The application server is built and developed by the user side and has different application functions for different IoT applications. Building a database to process and store the application data, providing an application logic interface, and using a visual interface such as WEB or APP to connect the user side with the application server mean building a LoRa IoT system from the sensing layer to the application layer.

### 3.2. Data-Driven Intelligent Law Classroom Analysis

Depending on the starting point for considering the problem, research scholars have proposed model-driven, goal-driven, product-driven, business-driven, technology-driven, decision-driven, task-driven, and other forms of drivers. Data analysis emanating from the model side is called model-driven, and the mode of operation with the technology side as the trigger is called technology-driven. With the advent of the intelligence and data era, knowledge acquisition is no longer entirely dependent on expert experience. The use of some intelligent methods can start from the data, mining to get the valuable information behind the data, which is data-driven and one of the main reasons why data-driven methods have developed more rapidly than other methods and attracted widespread attention from scholars [19]. From the perspective of the generation environment, the concept of data-driven is a product of the era of big data, inspired by data-intensive scientific discoveries. Data has become the starting point and perspective for people to understand, analyse and solve problems, resulting in the idea of using a data mindset to solve problems. Furthermore, the scientific preprocessing of data, the constant statistics of data, the real calculation of data, data modelling, machine learning, data visualization, data management, and other technologies provide the technical support for data-driven, in addition to these data science methods, data mining, neural networks, artificial intelligence, metadata, and all other technologies and methods of data-based design are the technical guarantee of data-driven. Reasonably use educational data to customize a personalized learning environment to meet the learning needs of students at different levels so that artificial intelligence and big data can truly promote more personalization, thereby increasing the attractiveness of the classroom:

(1) $$\begin{array}{c} T_{p}=\frac{\sqrt{x^{2}+y^{2}+z^{2}}}{C},\\ T_{s}=\frac{L}{V}-\frac{\sqrt{x^{2}-y^{2}-z^{2}}}{C}. \end{array}$$

**Figure 3:**
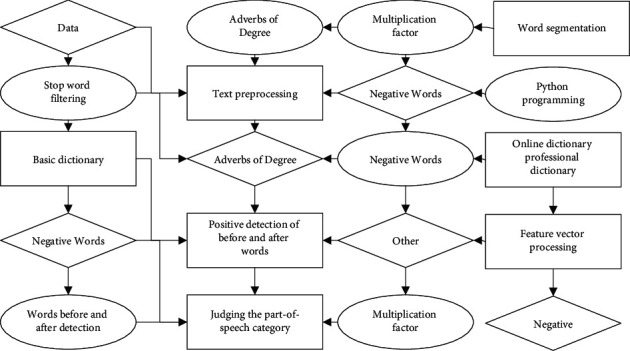
Data-driven intelligent law classroom architecture.

The essence and value of big data are to eliminate uncertainty, and the value of data drive lies in the kinetic output of the drive and the means to realize the value of big data: data analytic thinking, data-driven applications in different scenarios, the formation of data-driven decision-making, data-driven design, data-driven procedures, data-driven products, data-driven innovation, and other results output. Among them, the so-called data-driven decision-making is to help patronize through data, including product improvement, operation optimization, marketing analysis, and business decision-making. With data support, we can better determine which channels convert better and which feature styles are more popular with users and support the decision through data. Data-driven product intelligence is the ability to empower products to learn through data models. The so-called intelligence can be understood as a model with a certain database, setting an algorithm model on it; then, the results of the data obtained back to the product are intelligent. In this way, the product has a certain learning ability and can constantly update and iterate itself, for example, the literature recommendation system, by analysing the user's historical behaviour records, discovering the user's interest points, building a targeted user model, and finally realizing personalized recommendations, as shown in Figure 3.

Scientific discovery has shifted from the traditional hypothesis-driven to a scientific approach based on scientific data for exploration. For some scientific problems that are difficult for humans to understand, researchers are gradually unravelling the mystery of these scientific puzzles through data monitoring and analysis, using data as a tool and object of research and designing and conducting research from data. Data are no longer just the result of scientific research but have become the living foundation of scientific research. People are concerned with not only modelling, describing, organizing, preserving, accessing, analysing, reusing, and building scientific data infrastructure but also how to use the ubiquitous network and its inherent interactivity and openness to construct an open and collaborative discovery and creation model with the help of massive data, thus giving birth to data-intensive scientific discovery. There is a lack of effective and detailed information interaction between students and teachers. Teachers cannot specifically and comprehensively understand students' knowledge proficiency. They can only arrange teaching content and teaching progress based on their own teaching experience and random checks in the classroom. The development of a data-intensive scientific research paradigm offers new opportunities for collaborative efforts between academia, industry, and government. This research paradigm relies on sensing tools to acquire data or computer simulations to generate it, process it through software for cleaning, store it in computers, and analyse it using data analysis software. Data from scientific research can be grouped into four categories: data generated by experimental instruments, data generated by computer simulations, data collected by observation, and data generated on the Web. Data generated on the Web include historical behavioural data of Internet users and data on Web transactions:

(2) $$\begin{array}{c} a\left(h_{m}\right)=\left(1.25gf+0.7\right)h_{m}-\left(1.25gf-0.7\right),\\ P_{\text{coll},sf}=1+e^{2G_{sf}}. \end{array}$$

Data visualization is an interactive visual representation of data using the intelligence of the human brain and the perceptive ability of the human eye to convert data that is difficult to display directly into graphics, symbols, and so on to convey useful information efficiently. Through this visual representation of data, the information hidden in the data, including various attributes, is presented in some summary form. From the point of view of aesthetic requirements and functional requirements, data visualization requires the same for both and guarantees a balance between design and functionality by directly expressing special aspects and special features, and thus gaining insight into very sparse but particularly complex data collections.

The conflict model of the Lora WAN protocol shows that the probability of conflict during transmission of data packets sent by nodes using ALOHA access is closely related to the combination of bandwidth, coding rate, and the proportion of nodes using the same spreading factor. When the Lora WAN protocol is simulated in the LoRa network created in this paper, the corresponding proportion of nodes using the same spreading factor, bandwidth, coding rate, and other parameters are substituted into the formula, and the probability of conflicts occurring in the data transmission process of nodes using different spreading factors is close to 1. At this time, the conflicts in the network are more frequent, the data loss caused by communication conflicts is more serious, and the reliability of network communication is poor at this time:

(3) $$\begin{array}{c} G_{sf}=\lambda^{2}\partial_{sf}T_{sf}. \end{array}$$

**Figure 4:**
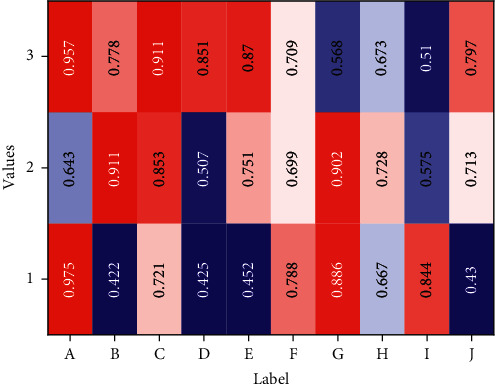
Label weights.

As the information transfer channel in the whole IoT architecture, the network layer is responsible for transferring the information obtained from the sensing layer and submitting it to the application layer for processing. The application layer is the interface between the IoT system and the user, which mainly solves the problems related to information interaction, human-machine processing, and so on. It combines the needs of various IoT industries to complete specific business functions and realize the intelligent application of the whole IoT industry. The main tasks of the application layer include data analysis and processing, data storage and mining, and communication handover between people and things, and things and things [20]. To adapt to the ubiquitous situation of devices and at the same time play the powerful computing ability, storage ability, and network communication ability of cloud computing platform, the cloud platform is used as the application layer in the IoT architecture to build the IoT cloud platform architecture; establish the unified management of people and things in the IoT application; establish and maintain the communication channel between people and things; and realize the functions of remote control, data management, business logic processing of industry applications, and so forth. The IoT system architecture based on the cloud platform is shown in Figure 4, which mainly consists of terminal layer, network access layer, and cloud platform.

For aesthetic effect, adjust the label size to be <10 so that the information about the research user can be better presented. The base feature is more important in understanding the researcher's information and is also the least variable. Set the base feature label size to 10. It is necessary to research and explore the intelligence and informatization of laboratories. This article takes the Computer Engineering Department of Fujian Institute of Information Technology as an example. Based on the existing facilities, the structure and characteristics of the Internet of Things technology in smart homes and smart buildings are applied to the laboratory management system:

(4) $$\begin{array}{c} i_{t}=\sigma \left(W_{i}\left|h_{t}x_{t}^{2}\right|+b_{i}\right). \end{array}$$

Research preference label and research relationship label: the user portrait of the digital library knowledge discovery platform designed in this paper is essentially a labeled formal description of the user model of the platform users, and the user portrait is constructed using the user's basic attributes, research results, and search target information and displayed through the word cloud visualization. Finally, the real users of the knowledge discovery platform are selected for the method validation. The user portrait of the digital library knowledge discovery platform constructed in this paper can depict user information in all aspects and intuitively display user information to accurately provide personalized services and accurate recommendations and provide a real and effective reference basis for the decision-making layer.

## 4. Results and Analysis

### 4.1. IoT Smart Classroom Teaching System Performance

The key to achieving remote management from the cloud to the device side is to establish and maintain instantaneous and reliable communication between the cloud and the device side. Instant communication requires that the device side can report data to the cloud, receive messages from the cloud in real time, and ensure that the communication has low latency. Since in the actual application environment, the network environment at the device side cannot ensure stability and reliability, so based on establishing the connection between the device side and the cloud side, a heartbeat mechanism is required to maintain the long connection from the device side to the cloud side, and at the same time, TCP-based protocols are preferred to select the communication protocols to ensure the reliability of the connection. Based on the above analysis, this paper selects the MQTT protocol to solve the problem of end-to-end instant and reliable communication during remote communication. MQTT protocol is a lightweight, agent-based publish/subscribe model messaging protocol designed for communication of remote sensors and control devices with limited computing and storage resources and working in low bandwidth and unreliable networks, with features of simplicity, scalability, and traffic saving. The MQTT protocol enables network communication between multiple clients by specifying topic subscriptions and message distribution, with the server acting as a proxy to forward messages based on the topic number. By decoupling the senders and receivers of messages in space and time, the system has good scalability and scaling capability.

The rapid development of IoT applications has led to the emergence of various types of IoT products. In various IoT products, the nodes can be either sensing devices/actuation devices with sensing data collection modules and control modules or subsystem devices connected through field buses. By accessing different functional module devices, different IoT products can be quickly constructed to achieve functional expansion of IoT applications. For the characteristics of multiple and complex product functions in IoT applications, this paper completes the description of device functions by defining attributes, services, and events for devices, referred to as the object model, to construct data models of device entities under different products, digitize the entity objects in physical space, and digitally represent them in the cloud, as shown in Table 1 for the definition of attributes and services events. By linking the Internet of Things technology with industry technologies, various solutions are used to provide users with a good Internet of Things business experience to achieve a wide range of intelligent applications, so that people can truly feel the huge impact of the Internet of Things on real life.

**Figure 5:**
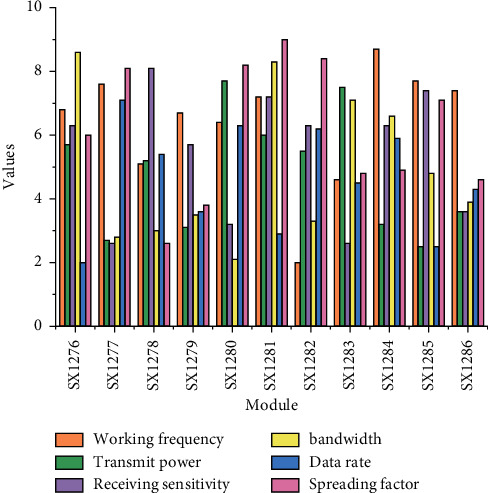
Comparison of the communication bloc.

Different IoT products are used in different application scenarios, and the hardware architecture and software environment of the devices in the products, as well as their functions, show great differences, which determine different forms and functions of the device side, leading to the gradual “fragmentation” of IoT applications. Any user-oriented IoT product needs to break the full-stack communication protocol of “device–network platform application,” but the “fragmented” IoT feature leads to great differences in the format, length, and precision of data from different devices. The communication protocols of different products are also very different, which will lead to the problem of interconnection between IoT products and become a bottleneck for further development of IoT. In the traditional IoT application development mode, first, the device developer needs to define the communication protocol from the device side to the application side according to the system function. The application side has a strong dependency on the device side development, so the device side must be done first and then the application side, which cannot be developed synchronously. The application developer must wait for the device developer to complete the development of the device side and then carry out the development and testing of the application side, which undoubtedly increases the development time and cost and consumes huge human and material resources, as shown in Figure 5. Data-driven decision-making, data-driven design, data-driven programs, data-driven products, and data-driven innovations are formed.

In order to avoid the communication conflict caused by multiple nodes communicating with the gateway at the same time on the same channel, CSMA presentation monitoring and random delay backoff strategy are used to avoid the conflict. Presend listening is to detect the presence of packet leading code on the channel through CAD. If the CAD detects the presence of leading code transmission in the current channel, then the current channel is occupied. Otherwise, the current channel is in an idle state. If the channel is idle, then the service transmission can be carried out. Otherwise, random back-off is performed. When the channel is occupied, the timer is set to wait for a random delay; after the delay, the current channel state is detected until the channel is idle and the data is sent successfully, or the number of random evasions reaches the upper limit. If the number of random evasions reaches the upper limit, the competing channel is randomly switched, and the data is sent again.

Since the SNR values of the three groups of nodes to the gateway are similar in this test, the gateway assigns the three nodes to the same TDMA channel after admission. They are assigned communication time slots consecutively in the order of admission. The three nodes continuously occupy the time slots for periodic service reporting. Use data as research tools and objects, design, and conduct research from data. Data is no longer just the result of scientific research but becomes the living foundation of scientific research. Theoretically, there is a difference of 3 seconds between the time slots of each node for periodic reporting. However, the test data shows that there is a delay of several tens of milliseconds due to the software and hardware factors. The corresponding communication cycle has a certain delay, but in general, it is verified that the LoRa network communication with TDMA access is feasible, and different nodes will actively report at different times without conflict with each other to ensure the reliability of communication is ensured.

### 4.2. Results of the Data-Driven Smart Law Classroom Experiment

**Figure 6:**
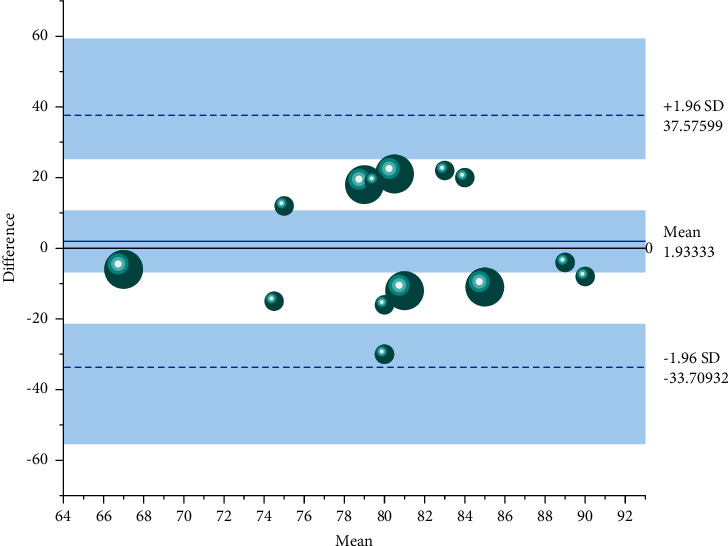
Test performance statistics.

Finally, classroom observations revealed a strong interest in the course, most notably in the reluctance of students to leave the classroom after class and continue to modify the code to create their work. Several problems were also revealed: firstly, the lack of basic computer skills due to the low exposure to computers, which was a major obstacle to the completion of the first three weeks of the assignment, and secondly, the repeated shouting at the teacher during the class. In the teacher workshop after the class, we analysed two reasons behind this: students were afraid of using the software badly because they were not familiar with the use of Scratch programming, so they were afraid to try boldly when there were problems with the code; students needed the teacher to affirm the quality of their work and encourage and evaluate it before they would proceed to the next stage of the task after completing their work. Show it in some form of summary. From the perspective of aesthetic requirements and functional requirements, the requirements of data visualization are the same for both, and by directly expressing special aspects and special characteristics, we can gain insight into very sparse but particularly complex data sets to ensure balance between design and function. The data collected was divided into three parts: the first part was about mathematical achievement, evaluated with a pre- and posttest of mathematical academic achievement related to project progress; the second part was computational thinking, divided into three areas (computational concepts and computational practices were evaluated by Bibras competition questions, computational perspectives were tested by scales, and collaborative perceptions in computational perspectives were analysed separately); and the third area was a problem-driven learning environment perceptual analysis, as shown in Figure 6.

**Figure 7:**
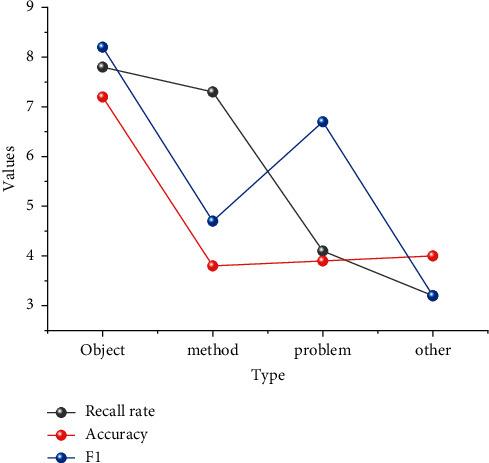
Comparison of inspection results.

Use the template to design a display page for the new seasonal products of Sun King Sportswear. According to the web design imitation + microinnovation concept, students can imitate the teacher's work or modify their preclass work. Moreover, according to their actual learning situation, they can choose a learning method that suits them; for example, students with weaker abilities can log on to the Super Star platform to watch tutorials and microlessons on demand and dialogue with robot assistants to help them learn, keep up with the pace of the class, and exercise their independent inquiry skills. Students upload the designed layout in real time to an online discussion group to exchange ideas with group members. This session uses online resources and a robot assistant to assist students in their task exploration, exercising their independent inquiry skills. Students discuss the layout online to improve their collaborative learning skills. The “shake it up” spot check of task completion adds interest to the classroom and instantly determines the level of proficiency in key content, recording student errors for more intuitive analysis and summary. The information resources include Super Star Online Learning Space, Turing Robot, Learning Pass “Shake One,” and Super Star Interactive Group. After the teacher's lecture, students can access the online learning space to learn more or ask questions to the robot assistant according to their proficiency. In this session, students can access the Superstar platform video to watch the learning data and discussion, and the students' personalized learning mode is greatly reflected. Online group interaction makes group learning more convenient. The use of “shake” instead of roll call to check the completion of tasks increases the interest of the classroom and improves students' participation in the classroom, as shown in Figure 7.

As can be seen from Figure 7, the accuracy of the research object is 0.90, the recall is 0.84, and the F1 value is 0.87. The research object category is higher than the other categories in all indicators of discrimination because the research object generally has an established authorized name. For example, in the case of research objects in specific feature domains, the corresponding conventions can usually be found, and the model can better identify and determine them. Research questions and research methods, on the contrary, are more variable and complex in their formal composition, so the discriminative F1 values are relatively low. The change of knowledge discovery services in data-driven digital libraries has put forward higher requirements on the semantic understanding of data resource contents, especially the full text of scientific research papers. Complete specific business functions and realize intelligent applications in the entire Internet of Things industry. The main tasks of the application layer include data analysis and processing, data storage and mining, communication between people and things, and things and things. To assist in achieving semantic identification, annotation, and understanding of data resource content, this section designs a keyword-based approach to identify the problems, methods, and research objects of data resource content using keywords of full-text scientific research papers and implements the approach in the field of agriculture. Experiments show that the method achieves satisfactory identification results. This section focuses only on identifying the research object, problem, and method vocabulary, without exploring the functions assumed by the vocabulary in the text and whether the problem corresponds to the method. Therefore, future research will further explore which specific function the vocabulary assumes in the text and what the corresponding solution to the problem is. Through the experiments, the effectiveness of the deep learning model is verified, which provides an effective general idea for the research design fingerprint extraction of the full text of scientific papers in the knowledge discovery service platform of the digital library and methodological references for other types of data content extraction and identification studies.

## 5. Conclusion

The LoRa IoT system is designed with the help of IoT platform, the access process from the device side to the cloud side is developed, and the authentication mechanism is used to ensure the legal access of the device side. The front-end web page of the data management website design is not beautiful enough, and the functions are not perfect. The jQuery and Bootstrap frameworks used in developing the front-end of the web page are not beautiful enough, and the website currently only does some simple data processing and visualization. Now, it does not support the function of big data analysis. The MQTT protocol is used to achieve reliable remote long connection communication between the cloud and the device; the device function is abstracted through the object model, and the object model is described using the JSON data interaction format so that the relevant communication protocol can be developed to realize the data transmission between the end and the end. The wireless communication-based classroom interaction system designed in this paper can help teachers realize the collection of feedback data, data visualization display and storage of students' roll call, multiple-choice questions of classroom quizzes, and Y/N judgment questions in classrooms. The data management website also allows students and teachers to view the actual classroom performance of students in their class or the teacher's class. Although the system can achieve the basic functions of the classroom interactive system, due to the limited personal ability, there are still numerous shortcomings, and there are some gaps between the research work of the thesis and the actual use of the needs, which need to be continuously improved at a later stage.

## Figures and Tables

**Table 1:** Properties, events, and service definitions.

Project	Describe	Value
Attributes	Parameters describing the operating state of the equipment	13
Service	The current temperature and humidity values collected by the temperature and humidity sensing device	14
Event	The attributes of the device support read-only, write-only, read and write, and users can read and set them	54

## Data Availability

The data used to support the findings of this study are available from the corresponding author upon request.

## References

[1] Kwet M., Prinsloo P. (2020). The “smart” classroom: a new frontier in the age of the smart university. *Teaching in Higher Education*.

[2] Engin Z., van Dijk J., Lan T. (2020). Data-driven urban management: mapping the landscape. *Journal of Urban Management*.

[3] Hillman V., Ganesh V. (2020). Kratos: a solution for data privacy, literacy, and student agency in a data driven school ecosystem. *International Journal of Innovation in Education*.

[4] Bennett A. (2021). Autonomous vehicle driving algorithms and smart mobility technologies in big data-driven transportation planning and engineering. *Contemporary Readings in Law and Social Justice*.

[5] Dong Z. Y., Zhang Y., Yip C., Swift S., Beswick K. (2020). Smart campus: definition, framework, technologies, and services. *IET Smart Cities*.

[6] Shahat Osman A. M., Elragal A. (2021). Smart cities and big data analytics: a data-driven decision-making use case. *Smart Cities*.

[7] Hong S. Y., Hwang Y. H. (2020). Design and implementation for iort based remote control robot using block-based programming. *Issues in Information Systems*.

[8] Coatney K., Poliak M. (2020). Cognitive decision-making algorithms, internet of things smart devices, and sustainable organizational performance in Industry 4.0-based manufacturing systems. *Journal of Self-Governance and Management Economics*.

[9] Petrovic N., Kocic D. (2020). Data-driven framework for energy-efficient smart cities. *Serbian Journal of Electrical Engineering*.

[10] Edwards C. (2021). Real-time advanced analytics, automated production systems, and smart industrial value creation in sustainable manufacturing internet of things. *Journal of Self-Governance and Management Economics*.

[11] Cerquitelli T., Pagliari D. J., Calimera A. (2021). Manufacturing as a data-driven practice: methodologies, technologies, and tools. *Proceedings of the IEEE*.

[12] Nguyen D. C., Cheng P., Ding M., Lopez-Perez D. (2020). Enabling AI in future wireless networks: a data life cycle perspective. *IEEE Communications Surveys & Tutorials*.

[13] Lucic M. C., Wan X., Ghazzai H., Massoud Y. (2020). Leveraging intelligent transportation systems and smart vehicles using crowdsourcing: an overview. *Smart Cities*.

[14] Zhang Y., Dong Z. Y., Yip C., Swift S. (2020). Smart campus: a user case study in Hong Kong. *IET Smart Cities*.

[15] Mihaljevic H., Larsen C. J., Meier S., Nekoto W., Morón Zirfas F. (2021). Privacy-centred data-driven innovation in the smart city. Exemplary use case of traffic counting. *Urban, Planning and Transport Research*.

[16] Schneider B., Reilly J., Radu I. (2020). Lowering barriers for accessing sensor data in education: lessons learned from teaching multimodal learning analytics to educators. *Journal for STEM Education Research*.

[17] Chen G., Wang P., Feng B., Li Y., Liu D. (2020). The framework design of smart factory in discrete manufacturing industry based on cyber-physical system. *International Journal of Computer Integrated Manufacturing*.

[18] Sarker I. H., Hoque M. M., Uddin M. K., Alsanoosy T. (2021). Mobile data science and intelligent apps: concepts, AI-based modeling and research directions. *Mobile Networks and Applications*.

[19] She C., Dong R., Gu Z. (2020). Deep learning for ultra-reliable and low-latency communications in 6G networks. *IEEE Network*.

[20] Rehm G. B., Woo S. H., Chen X. L. (2020). Leveraging IoTs and machine learning for patient diagnosis and ventilation management in the intensive care unit. *IEEE Pervasive Computing*.

